# Alcohol’s Effect on Host Defense

**DOI:** 10.35946/arcr.v37.2.01

**Published:** 2015

**Authors:** Gyongyi Szabo, Banishree Saha

**Affiliations:** Gyongyi Szabo, M.D., Ph.D., is professor and vice chair for research in the Department of Medicine, associate dean for clinical and translational research, and director of the M.D./Ph.D. Program; and Banishree Saha, Ph.D., is a senior postdoctoral research associate at the University of Massachusetts Medical School, Worcester, Massachusetts.

**Keywords:** Alcohol effects and consequences, alcohol consumption, alcohol exposure, acute alcohol exposure, chronic alcohol exposure, alcohol use pattern, alcoholic liver disease, pancreatitis, immunity, immune system, immune response, innate immune response, adaptive immune response, immunosuppression, bacterial disease, viral disease, inflammatory response, proinflammatory response, anti-inflammatory, infection, inflammation

## Abstract

Alcohol affects many organs, including the immune system, with even moderate amounts of alcohol influencing immune responses. Although alcohol can alter the actions of all cell populations involved in the innate and adaptive immune responses, the effect in many cases is a subclinical immunosuppression that becomes clinically relevant only after a secondary insult (e.g., bacterial or viral infection or other tissue damage). Alcohol’s specific effects on the innate immune system depend on the pattern of alcohol exposure, with acute alcohol inhibiting and chronic alcohol accelerating inflammatory responses. The proinflammatory effects of chronic alcohol play a major role in the pathogenesis of alcoholic liver disease and pancreatitis, but also affect numerous other organs and tissues. In addition to promoting proinflammatory immune responses, alcohol also impairs anti-inflammatory cytokines. Chronic alcohol exposure also interferes with the normal functioning of all aspects of the adaptive immune response, including both cell-mediated and humoral responses. All of these effects enhance the susceptibility of chronic alcoholics to viral and bacterial infections and to sterile inflammation.

Alcohol has been the most common substance of use and abuse in human history. Moderate amounts of alcohol are enjoyed for its anxiolytic effects; however, its addictive properties can lead to chronic, excessive alcohol use and alcohol use disorder. In addition to its commonly recognized behavioral effects, alcohol affects many organs, including the immune system that controls the body’s defense against infectious pathogens (e.g., bacteria and viruses) and other harmful agents. Chronic alcohol use is associated with significant alterations in the immune system that predispose people to viral and bacterial infections and cancer development. In general, severe chronic alcoholics are considered immunocompromised hosts. Although moderate alcohol use has less obvious clinical effects on the immune system, both in vitro and in vivo studies indicate that even moderate amounts of alcohol and binge drinking modulate host immune responses.

This review gives a general overview of the immune effects of alcohol. However, it is important to realize that many aspects of alcohol consumption and its effects on immunity and host defense have not yet been fully elucidated. For example, the pattern of alcohol consumption (e.g., occasional binge drinking versus chronic heavy drinking) may affect the immune system in different ways that are yet to be explored.

## Overview of the Immune System

The immune system serves to defend the host from pathogens and to prevent unwanted immune reactions to self. This defense involves coordinated complex interactions between two arms of the immune system—the innate and the adaptive immune responses. Innate immunity provides immediate responses to pathogen-derived or nonpathogen–associated (i.e., sterile) danger signals and results in activation of proinflammatory cytokines and/or Type I interferons, regardless of the underlying cause and without the body having encountered the pathogen before. Adaptive immunity, in contrast, which only sets in after a certain delay, is specific to the pathogen or antigen and requires an initial encounter with the pathogen or antigen to activate the response.

The innate immune response usually involves inflammatory reactions and/or production of reactive oxygen species (ROS) and other signaling molecules. Once the pathogen is eliminated, the innate immune response is resolved, allowing restoration of immune homeostasis (Miyake and Kaisho 2014; Mogensen 2009; Newton and Dixit 2012). The key cell types in innate immunity are a variety of white blood cells, including neutrophils, monocytes/macrophages, dendritic cells (DCs), and natural killer (NK) cells (table 1). In addition, the innate immune system has various soluble components, including the following:

- Cytokines, such as interleukin-(IL-)1, IL-6, or tumor necrosis factor alpha (TNFα), are produced by innate immune cells as part of the initial response and induce and support a full-fledged inflammatory response.
- Interferons mainly are produced by virus-infected cells and can induce an antiviral response in neighboring cells. Based on the receptors with which they interact they can be divided into three classes (Type I to Type III), of which the Type I interferons primarily are involved in the antiviral response.
- The complement system, a group of small proteins that mainly are produced in the liver and then released into the blood, recognize specific molecules on the surface of pathogens and help other immune molecules (i.e., antibodies) and immune cells (i.e., phagocytic cells) to identify and eliminate the pathogens from the organism. Thus, complement molecules can cover the pathogen in a process called opsonization, which enhances phagocytosis of the pathogen; attract macrophages and neutrophils to the pathogen; help rupture the membranes of foreign pathogens, and induce clustering and binding of pathogens.

The innate immune system is activated when the involved cells recognize certain immune danger signals. This recognition occurs through molecules called pattern recognition receptors, which include Toll-like receptors (TLRs), helicase receptors, and Nod-like receptors (NLRs).^1^ These receptors are strategically located on the cell surface or within the cells, where they can sense pathogen-derived signals, such as certain proteins, bacterial products called lipopolysaccharides (LPS), peptidoglycans, DNA, RNA, and numerous metabolic or other signals (Kawai and Akira 2009; Meylan et al. 2006; Seki and Brenner 2008). TLRs, NLRs, and helicase receptors are expressed on innate immune cells as well as on the functional cells (i.e., parenchymal cells) in most organs; however, activation of pattern recognition receptors triggers proinflammatory cytokine induction most robustly in the immune cells.

In addition to producing proinflammatory cytokines, innate immune cells (particularly DCs and monocytes) are necessary to present pathogen-derived molecules (i.e., antigens) to adaptive immune cells so as to trigger or facilitate adaptive immune responses. These adaptive immune cells include T cells, B cells, and natural killer T cells (NKTs), which must cooperate in a controlled manner to mount an effective response (Castellino and Germain 2006; Mitchison 2004). T cells in turn fall into several different categories, including helper T cells, also known as CD4^+^ cells; cytotoxic T cells, also called CD8^+^ cells; Th17 cells; and regulatory T (Treg) cells (table 1). As the name implies, helper T cells help control the activity of other immune cells by producing and secreting various cytokines.

Depending on the specific cytokines they produce, helper T cells can further be subdivided into two types with specific functions:

- Th1 cells, which initiate a cell-mediated immune response against intracellular pathogens by activating macrophages or cytotoxic T cells that then destroy the pathogen. The Th1 cells primarily induce their effects by releasing interferon gamma (IFN-γ), which promotes inflammatory responses.
- Th2 cells, which initiate a humoral immune response against extracellular pathogens that is mediated by proteins (i.e., immunoglobulins [Igs]) produced by B cells. The Th2 cells induce their effects primarily by releasing a variety of interleukins, some of which have anti-inflammatory effects.

Th17 cells also can be considered a type of helper T cells characterized by the production of interleukin 17. Their main function is to defend against pathogens at epithelial and mucosal barriers. Finally, Treg cells serve to limit and suppress the immune response to prevent overreaction of the immune system as well as immune reactions against self-antigens.

B cells are characterized by the production of antibodies comprised of Igs. Various types of Igs (e.g., IgA, IgG, IgM) are produced at different times during an infection or in response to a range of antigens that have specific roles in the adaptive immune response.

In contrast to the innate immunity, which can be induced by any kind of antigen, adaptive immune responses are specific to individual antigens. In other words, each T cell or B cell can be activated only by one specific antigen. An antigen-specific T-cell response is initiated by interactions between antigen presenting cells (such as DCs) and naïve T cells and is optimized by engagement of co-stimulatory molecules and cytokines for antigen-specific T-cell activation (Mogensen 2009; Newton and Dixit 2012). The initial activation triggers a memory response in the form of memory B cells that remain in the circulation for long periods and can respond quickly when they encounter that antigen a second time to mount a stronger, more rapid response.

The complexity of the innate and adaptive immune responses are increased further by the fact that different subsets of immune cells may reside in specific organs, such as the liver, lungs, brain, skin, bones, or muscles. This complex structure of the immune system with its multitude of different cells with diverse functions allows the organism to defend itself properly against the hugely diverse pathogens it may encounter, without endangering its own cells. At the same time, it makes it much more difficult to investigate and understand the impact of external influences, such as acute or chronic alcohol exposure, on the body’s immune responses.

## Alcohol’s Effects on the Immune System

Alcohol can modulate the activities of all of these cell populations by affecting the frequency, survival, and function of most of these cells, thereby interfering with pivotal immune responses. However, unlike other mechanisms that cause classical immunocompromised states, such as HIV or tuberculosis infection, alcohol use typically results in a subclinical immunosuppression that becomes clinically significant only in case of a secondary insult. For example, chronic alcohol consumption increases the risk and severity of chronic infections with HIV; hepatitis C virus (HCV); or *Mycobacterium tuberculosis*, the bacterium that causes tuberculosis, and promotes post-trauma immunosuppression (for more information, see the articles in this issue by Bagby and colleagues, by Dolganiuc, by Molina and colleagues, and by Simet and Sisson).

Emerging evidence also suggests that alcohol may affect immune functions by altering the balance and interactions between the host immune system and the entirety of microorganisms found in the host (i.e., the host microbiome). This microbiome is composed of the normal microorganisms found in and on the body (i.e., commensal microorganisms), which are needed for the body’s normal functioning, and disease-causing pathogens. Increasing evidence suggests that alcohol may modulate the composition of pathogenic and commensal organisms in the microbiome of the gut, oral cavity, skin, and other mucosal surfaces (Chen and Schnabl 2014; Leclercq et al. 2014*a*,*b*). These alcohol-induced changes could have clinical significance because the composition of the microbiome sends important pathogenic as well as homeostatic signals for the functions of host immunity. For example, chronic alcohol use is associated with changes in the gut microbiome, both increasing the microbial content in the first part of the large intestine (i.e., cecum) and changing the abundance of different types of microorganisms in the gut (Chen and Schnabl 2014; Fouts et al. 2012; Yan et al. 2011). This may alter the levels of LPS released by certain types of bacteria in the gut, which can contribute to inflammation in alcoholic liver disease as well as in liver cancer (i.e., hepatocellular carcinoma) (Chassaing et al. 2014; Gao et al. 2011; Szabo and Bala 2010). (For more information, see the articles in this issue by Hammer and colleagues and by Engen and colleagues).

### Alcohol and Innate Immunity

Alcohol modulates the function of nearly all components of the innate immune system, but the specific effects on inflammatory cell responses depend on the pattern of alcohol exposure (i.e., acute or chronic). In human monocytes or mouse macrophages, acute alcohol results in a decrease in TLR responses (i.e., TLR tolerance), which attenuates particularly production of the TNFα in response to a subsequent LPS stimulation (Bala et al. 2012; Mandrekar et al. 2009). However, this initial inhibitory effect of acute alcohol on monocytes and macrophages is transient, and repeated alcohol exposure (such as in chronic alcohol use) leads to loss of TLR4 tolerance; instead, the cells become more responsive to LPS stimulation, a process known as sensitization (Mandrekar and Szabo 2009; Mandrekar et al. 2009). Even a single episode of binge drinking can have measurable effects on the innate immune system, inducing a transient proinflammatory state within the first 20 minutes after alcohol ingestion, followed by an anti-inflammatory state 2 to 5 hours after alcohol ingestion (Afshar et al. 2015).

Both TLR tolerance and sensitization of monocytes and macrophages are associated with the production of specific sets of signaling molecules. The molecular signatures of alcohol-induced TLR tolerance and sensitization, respectively, have been well described and involve downstream components of the TLR-induced signaling cascades. This includes activation of molecules called IRAK-M, IRAK1/4, Bcl-3, and NF-κB, all of which regulate proinflammatory cytokine activation (Bala et al. 2012; Mandrekar and Szabo 2009; Mandrekar et al. 2009). (For a list of the full names of these and other molecules mentioned in this article, please see table 2.) The proinflammatory effect of prolonged alcohol exposure has been demonstrated in response to molecules (i.e., ligands) that activate TLR4, TLR3, and TLR2 receptors (Bird et al. 2010; Fernandez-Lizarbe et al. 2013; Goral and Kovacs 2005; Oak et al. 2006; Pruett et al. 2004).

It is increasingly evident that sensitization of proinflammatory pathways to activation in monocytes and macrophages after chronic alcohol use has biological and clinical significance. It is known that alcohol-mediated sensitization of immune cells to gut-derived LPS is a major component in the pathogenesis of alcoholic liver disease and alcoholic pancreatitis (Choudhry et al. 2002; Keshavarzian et al. 1994; Nolan 2010; Szabo et al. 2010, 2011). In fact, in acute alcoholic hepatitis, the severity of clinical outcome and death correlates with serum levels of the proinflammatory cytokines, particularly TNFα (Frazier et al. 2011; McClain et al. 2004). (For more information on the role of innate immunity in the pathogenesis of alcoholic liver disease, see the articles in this issue by Nagy and by Mandrekar and Ju.) Chronic alcohol use also promotes inflammation in the small bowel, brain, lungs, and other organs, suggesting that common mechanisms may underlie the proinflammatory effects of alcohol. The exact triggers for alcohol-induced inflammation in the different tissues are yet to be identified. Importantly, deficiency in TLR4, the major sensor of LPS, attenuates inflammation induced by chronic alcohol use in the liver, brain, and intestine (Hritz et al. 2008; Lippai et al. 2013*a*,*b*, 2014). However, LPS increase was not found in the brain, suggesting that other ligands and/or alcohol itself may activate TLR4 (Alfonso-Loeches et al. 2010; Lippai et al. 2013b).

In addition to direct induction of chemokines and most proinflammatory cytokines by TLR activation, activation of the inflammasome was detected in the liver, brain, and intestine after chronic alcohol use (Orman et al. 2013; Szabo and Lippai 2014). The inflammasome is a multiprotein intracytoplasmic complex that comprises a sensor (e.g., NLRP1, NLRP3, NLRC4, or a protein called AIM2) and adapter molecules (e.g., a molecule called ASC). This protein complex can be activated by a variety of sterile danger signals (Tsuchiya and Hara 2014). Activation of the inflammasomes results in induction of caspase-1, an enzyme needed to form mature secreted IL-1β or IL-18. Recent studies have demonstrated inflammasome activation and IL-1β induction in the liver, brain, and intestine after chronic alcohol administration in mice (Alfonso-Loeches et al. 2010; Lippai et al. 2013*a*,*b*, 2014; Orman et al. 2013). These findings are biologically significant, because administration of a recombinant IL-1 receptor antagonist that blocks signaling via the IL-1 receptor can attenuate alcohol-induced liver disease and cerebral inflammation (Petrasek et al. 2012). These observations demonstrate that chronic alcohol administration results in inflammation and leads to a vicious cycle of upregulation of the inflammatory cascade. Future studies are needed to evaluate whether disruption of this vicious cycle would be sufficient to attenuate and or prevent chronic alcohol-induced tissue damage in various organs.

Alcohol also interferes with the body’s normal mechanisms that help control the innate immune response and prevent excessive inflammatory reactions. These mechanisms include the induction of anti-inflammatory cytokines, such as IL-10 and transforming growth factor beta (TGFβ) (Ouyang et al. 2011; Sanjabi et al. 2009). Again, the specific effects depend on the duration of alcohol exposure. Thus, whereas acute alcohol exposure increases both IL-10 and TGFβ production in monocytes and macrophages, chronic alcohol exposure mostly is associated with decreased IL-10 production or prevents appropriate increases in IL-10 levels to counterbalance the overproduction of proinflammatory cytokines (Byun et al. 2013; Järveläinen et al. 1999; Mandrekar et al. 2006; Norkina et al. 2007; Pang et al. 2011).

Alcohol exposure may modify not only cytokine secretion but also the overall function of monocytes and macrophages. These cells exhibit remarkable plasticity that allows them to change their phenotype from a proinflammatory (M1) phenotype that inhibits cell proliferation and can cause tissue damage to an alternatively activated type (M2) that has anti-inflammatory and tissue-repair capacity (Italiani and Boraschi 2014). This process is known as polarization. Interestingly, in alcoholic liver disease, which would be expected to be characterized by the presence of primarily proinflammatory M1 macrophages, the numbers of both M1 and M2 macrophages are increased. An alcohol-induced shift toward M2-type cells may have some beneficial effects by destroying pro-inflammatory M1 macrophages (Wan et al. 2014); however, the fibrogenic effect of M2 macrophages that leads to the formation of scar tissue also can damage liver function. Further studies on the effects of alcohol on monocyte/macrophage polarization may reveal potential therapeutic interventions for alcohol-induced immunomodulation.^2^

Neutrophils represent another important innate immune cell type affected by alcohol. Studies found that alcohol increases ROS production by neutrophils; however, their phagocytic capacity, which is important in antibacterial defense, was decreased by alcohol administration (Gandhi et al. 2014; Karavitis and Kovacs 2011).^3^ Interestingly, recruitment of neutrophils to the liver is a characteristic of the pathology of acute alcoholic hepatitis. Recent studies suggested that the increase in the numbers of neutrophils in the liver correlates with survival in acute alcoholic hepatitis (Altamirano et al. 2014); however, the role of neutrophils in this process is not yet fully understood.

The pattern-recognition receptors (i.e., TLRs, NLRs, and helicase receptors) found on innate immune cells play a pivotal role particularly in the defense against viral infections. These receptors recognize viral nucleic acids (i.e., DNA and RNA) and mount an immediate response mediated by interferons (Stetson and Medzhitov 2006; Takeuchi and Akira 2009). Production of interferons in monocytes is induced by activation of various TLRs and helicase receptors. The actions of interferons within the cells, in turn, are mediated by regulatory molecules called signal transducers and activators of transcription (STATs), a family of transcription factors that regulate the expression of certain immune genes. Alcohol interferes with these processes at multiple levels. Thus, both acute and chronic alcohol inhibit induction of Type-I interferons via TLR3, TLR7/8, or TLR9 or by helicase receptors in monocytes (Pang et al. 2011; Pruett et al. 2004). Alcohol also impairs Type-I interferon-receptor signaling by inhibiting STAT signaling (Norkina et al. 2008; Plumlee et al. 2005).

### Alcohol’s Effects on Adaptive Immunity

Many studies have evaluated the effects of chronic alcohol on adaptive immune responses, and this research is reviewed in more detail in the article by Pasala and colleagues in this issue. Chronic alcoholics have impaired T-cell responses; moreover, the balance between Th1 and Th2 responses is shifted toward a predominance of Th2-type responses (Fan et al. 2011; Lau et al. 2006; Szabo 1999). Consistent with this, chronic alcoholics exhibit an increase in IgA and a relative decrease in IgG antibodies, which play a role in antibody-dependent cell-mediated immune responses (Massonnet et al. 2009; Nouri-Aria et al. 1986). Other studies have noted a greater-than-normal abundance of Th17 cells in people with alcoholic liver disease (Lafdil et al. 2010; Ye et al. 2011). Specific aspects of the adaptive immune response also are affected. For example, even a single dose of alcohol may impair antigen-specific T-cell activation. Thus, in human monocytes and myeloid DCs, alcohol inhibits the cells’ antigen-presentation function as well as their capacity to induce antigen-specific (Mandrekar et al. 2009) and general T-cell activation (Szabo et al. 2001).

## Alcohol’s Effects on Maturation and Development of Immune Cells From Precursors

Alcohol abuse has an adverse effect on hematopoiesis and can cause leukopenia, granulocytopenia, and thrombocytopenia in humans (Latvala et al. 2004). Acute alcohol can block differentiation or maturation of granulocytes (i.e., granulopoiesis) during infections (Zhang et al. 2009). Examination of the bone marrow from alcoholic patients has shown vacuolated granulopoietic progenitors with a significantly reduced number of mature granulocytes (Yeung et al. 1988). Alcohol intoxication also can suppress the myeloid proliferative response by inhibiting the Stem Cell Antigen-1/ERK pathway during bacterial infection (Melvan et al. 2012). Chronic alcohol consumption also affects the NKT cell populations that play important immunoregulatory roles. Thus, alcohol consumption enhances immature NKT (iNKT) cell proliferation and maturation in the thymus and increases IFN-γ–producing iNKT cells (Zhang et al. 2015). In vivo activation of iNKT cells induces a Th1-dominant immune response and enhances the activation of DCs as well as NK cells, B cells, and T cells in alcohol-consuming mice (Zhang et al. 2015).

DCs, which are the major cell type linking the innate and adaptive immune response, also are affected by alcohol intoxication. Acute alcohol exposure alters function and cytokine production in human monocyte-derived myeloid DCs (Szabo et al. 2004*a*). Chronic alcohol consumption in humans causes alterations in the immunophenotype of DCs and decreased production of IL-1β and TNFα (Laso et al. 2007). Studies in rhesus macaques have helped elucidate the effects of alcohol on DC development in hematopoietic tissues and the functional activities of the DCs (Siggins et al. 2009). In these studies, chronic alcohol exposure decreased the pools of myeloid DCs in the bone marrow and peripheral blood. Alcohol also suppressed expression of the co-stimulatory molecule CD83 during DC maturation, which may attenuate the ability of DCs to initiate T-cell expansion (Siggins et al. 2009).

## Alcohol-Induced Modulation of the Host Defense Against Different Pathogens

It has been known for decades that chronic alcoholic individuals have increased susceptibility to infections (Sternbach 1990; Szabo 1999). This increased susceptibility to both viral and bacterial infections has been attributed to alcohol’s general immunosuppressive effects, and animal models of chronic alcohol use and infections repeatedly have confirmed this (Jerrells et al. 1994, 2007). In addition, chronic alcoholics seem to be vulnerable to inflammatory reactions not associated with pathogenic infections (i.e., sterile inflammation).

**Glossary**

Antibody: Immune molecule (protein) produced by *B cells* that recognizes foreign molecules that have entered the body (i.e., *antigens*), binds to these molecules, and marks them for destruction by the body’s immune system
Antigen: Any molecule that can bind specifically to an *antibody* and can induce an immune response
B Cells: One of the two main types of lymphocytes involved in the adaptive immune response; when activated by interacting with a specific *antigen*, they differentiate into specific subtypes and begin to produce *antibodies* that recognize the specific *antigen*
Cell-Mediated Immune Response: Part of the adaptive immune response that is mediated by various populations of *T cells*
Chemokine: Small proteins that serve as chemoattractants, stimulating the migration and activation of cells, particularly phagocytic cells and lymphocytes; they have a central role in inflammatory responses
Cytokine: Any of a group of molecules, produced primarily by immune cells, that regulate cellular interactions and other functions; many cytokines play important roles in initiating and regulation inflammatory reactions
Dendritic Cell: A type of immune cell involved in the innate immune response that are characterized by a branched morphology; dendritic cells can bind to *antigens* and present these antigens to *T cells*, thereby initiating an adaptive immune response
Granulocytopenia: Condition in which the number or proportion of certain white blood cells (i.e., granulocytes) in the blood is lower than normal; granulocytes, which are characterized by the presence of small, enzyme-containing vesicles (i.e., granules) in the cytoplasm, are part of the innate immune system
Helicase Receptors: A class of proteins that act as intracellular pattern recognition receptors and play a central role in the innate immune system; they recognize the presence of viruses in the cells and initiate antiviral responses
Hematopoiesis: The entirety of the processes through which the different blood cells are formed
Humoral Immune Response: Part of the adaptive immune response that is mediated by various populations of *B cells* and the *antibodies* they produce
Inflammasome: A complex comprised of several proteins that is a component of the innate immune system and is responsible for activation of inflammatory processes; it promotes the maturation of several inflammatory *cytokines*
Leukopenia: Condition in which the number or proportion of white blood cells (i.e., leukocytes) in the blood is lower than normal
Macrophage: A type of immune cell that ingests foreign particles and micro-organisms in a process called *phagocytosis* and which synthesizes *cytokines* and other molecules involved in inflammatory reactions
Natural Killer (NK) Cell: A type of immune cell involved in the innate immune response that can kill certain harmful cells, particularly tumor cells, and contributes to the innate immune response to cells infected with viruses or other intracellular pathogens
Neutrophil: A type of immune cell involved in the innate immune response that engulfs and kills extracellular pathogens in a process called *phagocytosis*
Nod-Like Receptors (NLRs): A class of proteins that act as pattern recognition receptors and play a central role in the innate immune system; they are embedded in the membrane of various immune and nonimmune cells and can recognize certain bacterial molecules, thereby initiating the immune response to the bacteria
Opsonization: The process by which *antibodies* bind to a pathogen, thereby marking it for destruction by *phagocytosis*
Parenchymal Cells: The cells in an organ that comprise the functional part of the organ (e.g., the hepatocytes in the liver)
Phagocytosis: Internalization or engulfment of particles or cells by specific cells (i.e., phagocytes), such as such as *macrophages* or *neutrophils*
T Cells: One of the two main types of lymphocytes involved in the adaptive immune response after activation through the interaction with a specific *antigen*. T cells can be divided into several subgroups that support other immune cells (helper T cells), kill invading pathogens or infected cells (cytotoxic T cells), or help turn off the adaptive immune response (regulatory T cells)
Thrombocytopenia: Condition in which the number or proportion of platelets (i.e., thrombocytes) in the blood is lower than normal
Toll-Like Receptors (TLRs): A class of proteins that act as pattern recognition receptors and play a central role in the innate immune system; they are embedded in the membrane of *macrophages* and *dendritic cells* and can recognize molecules derived from pathogens, thereby initiating the immune response to those pathogens

### Viral Infections

Most evidence for alcohol-associated increases in susceptibility to infection comes from studies of human viral infections, such as HCV, hepatitis B virus (HBV), HIV, and pulmonary viral infections. Such investigations have yielded the following findings:

- The prevalence of HCV infection is higher in individuals with chronic alcohol use than in the general population. Alcohol exposure and HCV interact at several levels. For example, alcohol exposure augments HCV replication by altering the levels of a molecule that supports HCV replication (i.e., microRNA-122) in liver cells (i.e., hepatocytes) (Hou et al. 2013). Moreover, alcohol and HCV synergistically impair antiviral immunity by interfering with the function of antigen-presenting cells, altering the activity and frequency of Treg cells, and modifying production of Type-I interferons (Dolganiuc et al. 2003; Plumlee et al. 2005; Szabo et al. 2004*b*). In patients with liver disease caused by chronic HCV infection, chronic alcohol use is an independent risk factor for development of advanced liver disease and cirrhosis (Corrao and Arico 1998; Szabo 2003).
- Chronic HBV infection affects about 240 million people worldwide (Centers for Disease Control and Prevention 2013). Research has shown that alcohol use accelerates the progression of liver disease caused by chronic HBV infection to liver fibrosis and hepatocellular cancer (Gao and Bataller 2011; Zakhari 2013). However, the cellular and molecular mechanisms by which alcohol and HBV interact still await further investigations.
- Studies on the effect of alcohol on HIV infectivity in humans have yielded conflicting results. However, the combined immunosuppressive effects of alcohol use and advanced HIV infection (AIDS) are well established (Muga et al. 2012; Szabo and Zakhari 2011).
- In pulmonary viral infections, it is unclear whether alcohol increases susceptibility to influenza infections or adversely affects the outcome of established infections. However, in animal models of pulmonary infections, alcohol administration is associated with adverse clinical parameters and increased lung damage (Boé et al. 2009; Zhang et al. 2008).

### Bacterial Infections

Bacterial infections can be either systemic or localized to a specific organ, such as the lungs. Alcohol use has negative effects on all types of pulmonary bacterial infections. For example, infections with *Mycobacterium tuberculosis* are more severe in chronic alcoholics, and alcohol use is associated with systemic dissemination of tuberculosis (Rehm et al. 2009; Szabo 1997). Furthermore, infections with *Klebsiella pneumoniae* and *Streptococcus pneumoniae*, common causes of pneumonia in humans, are more common in alcoholics compared with the nonalcoholic general population (Bhatty et al. 2011; Jong et al. 1995). Alcohol-induced dysfunction of specific immune cells contributes to severe pneumonias in this population. For example, the function of alveolar macrophages is impaired because of alcohol-induced changes in cytokine profiles as well as in the levels of ROS and antioxidants that result in oxidative stress (Liang et al. 2012; Mehta and Guidot 2012). Recruitment and function of neutrophils in alcoholic individuals also are increased, resulting in increased tissue damage in the lung alveoli (Dorio and Forman 1988; Kaphalia and Calhoun 2013).

Not only chronic alcohol abuse but also acute alcohol exposure can impair immune response to pulmonary infections. For example, acute intoxication in humans with blood alcohol levels of 0.2 percent can severely disrupt neutrophil functioning and their ability to destroy bacteria (Tamura et al. 1998). Studies in laboratory animals have confirmed the adverse effects of acute alcohol exposure on pulmonary infections. Thus, acute alcohol exposure in animals that were then infected with *S. pneumoniae* impaired lung chemokine activity in response to the infection, which resulted in reduced recruitment of immune cells into the lungs, decreased bacterial clearance from the lungs, and increased mortality (Boé et al. 2001; Raasch et al. 2010). The effects of both acute and chronic alcohol exposure on the immune responses in the lungs and thus on susceptibility to pulmonary infections are discussed in more detail in the article by Simet and Sisson.

### Sterile Inflammation

Inflammatory reactions (i.e., innate immune responses) can be induced not only by invading pathogens but also by danger signals resulting from damage to the body’s own cells. Elucidation of the immune processes occurring in response to damaged self also may offer a better understanding of the proinflammatory effects of alcohol in various organs (e.g., liver or brain). One example of this is the relationship between gut-derived bacterial LPS, alcohol exposure, and inflammatory reactions. Although gut-derived LPS clearly has a role in alcoholic liver disease, it is equally clear that LPS alone does not cause alcoholic liver disease. Many other conditions associated with increased levels of gut-derived LPS in the systemic circulation, such as HIV infection or inflammatory bowel disease, do not involve liver disease (Caradonna et al. 2000; Marchettia et al. 2013). Furthermore, inflammatory reactions can occur in the brain after alcohol use, even in the absence of detectable LPS in the brain (Lippai et al. 2013*a*; Szabo and Lippai 2014). These observations suggest that although gut-derived LPS can promote tissue inflammation, another alcohol-induced component is required as well. Thus, it seems that alcohol exposure directly leads to the release of sterile danger signals from parenchymal cells in different tissues, which in turn result in the activation of inflammatory cells via TLRs and NLRs. These alcohol-induced sterile danger signals include a wide variety of molecules, such as high-mobility-group protein B1 (HMGB1), heat shock proteins, adenosine triphosphate (ATP), and potassium ions (Rock and Kono 2008; Sangiuliano et al. 2014).

It is now thought that alcohol-induced sterile danger signals contribute to the proinflammatory cytokine activation seen after chronic alcohol use in various organs (e.g., liver, intestine, and brain). This hypothesis also is supported by findings that in hepatocytes, alcohol exposure results in a rapid induction of apoptosis, which precedes induction of inflammatory cytokines (Caradonna et al. 2000; González-Reimers et al. 2014; Marchettia et al. 2013; Petrasek et al. 2013). Additional evidence for the role of sterile inflammatory signals in alcohol-induced inflammation and tissue damage comes from findings that HMGB1 is increased both in the liver and brain after chronic alcohol exposure (Crews et al. 2013; Csak et al. 2014; Lippai et al. 2013*a*,*b*). Finally, NLRs, specifically NLRP3 and NLRP4, have been found to be involved in alcoholic liver inflammation. Given the role of NLRs in sensing endogenous danger molecules, this observation further supports the notion that alcohol-induced tissue inflammations is caused at least partially by alcohol-induced danger signals.

## Summary

As this review has indicated, alcohol exposure, and particularly chronic alcohol use, has profound effects on all aspects of the body’s immune responses, including both innate and adaptive immunity. These effects can impair the body’s defenses against a wide range of pathogens, including viruses and bacteria, as well as against damaged self and can affect tissues and organs throughout the body (see the figure). Thus, alcohol’s effects on innate immune responses seem to promote inflammatory reactions, which may contribute to tissue damage in a variety of organs. Alcohol-related impairments of adaptive immune responses render the organism more vulnerable to viral and bacterial infections, contributing to more severe or accelerated disease progression. In addition, dysregulation of normal immune responses may contribute to such conditions as alcoholic liver disease and pancreatitis, altered gut permeability and gastrointestinal inflammation, neuroinflammation in the brain, and the development of cancer (see the article by Meadows and Zhang).

The following articles in this journal issue look at various aspects of alcohol’s impact on innate and adaptive immune responses in more detail. They will also further explore the consequences of alcohol-induced disturbances of immune function on a variety of specific conditions, including liver disease, lung disease, cancer, traumatic injury, and bacterial and viral infections. Together, these articles will highlight the pivotal role that alcohol’s effects on immune function play in the overall morbidity and mortality associated with excessive alcohol use.

## Figures and Tables

**Figure:**
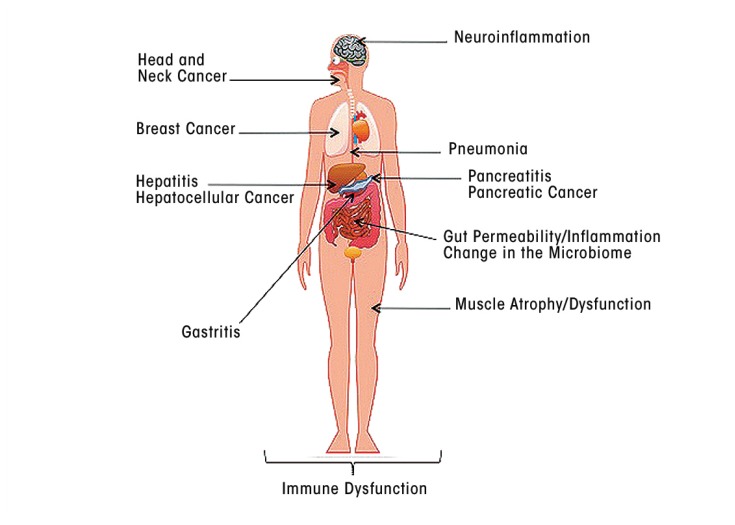
Overview of alcohol’s effects on human health that are associated with alcohol-induced dysfunction of the immune system.

**Table 1:** Cells Involved in the Innate and Adaptive Immune Responses

Cell Type	Characteristics	Functions
**Innate Immune Responses**		
Dendritic Cells	Have a roughly star-shaped form with several arms	Present antigens to other immune cells Stimulate T-cell responses Produce interferons (IFNs) Produce cytokines and reactive oxygen species (ROS)
Monocytes/Macrophages	Have a kidney-shaped nucleus Monocytes are precursors of macrophages; mature into macrophages when they enter the tissues Specific subtypes of macrophages reside in the tissues (Kupffer cells in the liver, microglial cells in the brain, alveolar macrophages in the lungs)	Destroy pathogens by phagocytosis Produce cytokines and ROS
Natural Killer (NK) Cells		Destroy cells infected with viruses and intracellular pathogens
Neutrophils	Have a multi-lobed nucleus	Destroy pathogens by phagocytosis Produce ROS

**Adaptive Immune Response**		
CD4 T Cells	Express CD4 glycoprotein on their cell surface	Helper T cells that activate B cells and macrophages
Th1 Cells	Primarily act by secreting IFN gamma (IFNγ)	Initiate a humoral immune response; have some anti-inflammatory effects
Th2 Cells	Primarily act by secreting various interleukins	Initiate cell-mediated immune response; have proinflammatory effects
Th17 Cells	Subtype of CD4 T cells Produce interleukin (IL)-17	Involved in recruitment, activation, and migration of neutrophils Provide defense against pathogens at mucosal and epithelial barriers
CD8 T Cells	Express CD8 glycoprotein on their cell surface	Cytotoxic T cells Destroy virus-infected and tumor cells
Treg Cells	Formerly known as suppressor T cells	Inhibit T-cell responses to prevent excessive immune reactions
B Cells		Produce antibodies Form memory cells
NKT Cells	Share properties of NK cells and T cells	Produce multiple cytokines Can perform functions ascribed to both helper and cytotoxic T cells

**Table 2:** Full Names of Molecules Mentioned in the Article

AIM2	Absent in melanoma 2
ASC	Apoptosis-associated speck-like protein containing a CARD
Bcl-3	B-cell lymphoma 3-encoded protein
ERK	Extracellular signal–regulated kinase
IFN-γ	Interferon gamma
Ig	Immunoglobulin
IL	Interleukin
IRAK1/4	Interleukin-1 receptor–associated kinase 1/4
IRAK-M	Interleukin-1 receptor–associated kinase M (restricted to monocytes/macrophages)
LPS	Lipopolysaccharide
NF-κB	Nuclear factor “kappa-light-chain-enhancer” of activated B cells
NLR	Nod-like receptor
NLRP	Nod-like receptor, subfamily P
STAT	Signal transducer and activator of transcription
TGFβ	Transforming growth factor beta
TLR	Toll-like receptor
TNFα	Tumor necrosis factor alpha

## Footnotes

1 NLRs can be classified into four subfamilies based on their specific structure at one end of the molecules. These families are called NLRA, NLRB, NLRC, and NLRP. Each of these subfamilies may comprise several members (e.g., NLRP1, NLRP2, etc.).

2 Recent studies indicate that the distinction between M1 and M2 macrophage populations may not be as clear cut because the markers that have delineated these two populations have become blurred and because macrophage polarization falls along a spectrum between the historic M1 and M2 phenotypes (Martinez and Gordon 2014; Murray et al. 2014; Xue et al. 2014). Thus, the concept of macrophage polarization currently is an evolving area.

3 Alcohol alters not only phagocytosis mediated by neutrophils but also phagocytosis by macrophages.
